# Application of Tandem Two-Dimensional Mass Spectrometry for Top-Down Deep Sequencing of Calmodulin

**DOI:** 10.1007/s13361-018-1978-y

**Published:** 2018-06-04

**Authors:** Federico Floris, Lionel Chiron, Alice M. Lynch, Mark P. Barrow, Marc-André Delsuc, Peter B. O’Connor

**Affiliations:** 1 Department of Chemistry, University of Warwick, Coventry, CV4 7AL UK; 2 CASC4DE, 20 Avenue du Neuhof, 67100 Strasbourg, France; 3 Institut de Génétique et de Biologie Moléculaire et Cellulaire, Institut National de la Santé et de la Recherche, U596; Centre National de la Recherche Scientifique, Unité Mixte de Recherche 7104, Université de Strasbourg, 67404 Illkirch-Graffenstaden, France

**Keywords:** FT-ICR MS, 2DMS, Top-down proteomics

## Abstract

Two-dimensional mass spectrometry (2DMS) involves simultaneous acquisition of the fragmentation patterns of all the analytes in a mixture by correlating their precursor and fragment ions by modulating precursor ions systematically through a fragmentation zone. Tandem two-dimensional mass spectrometry (MS/2DMS) unites the ultra-high accuracy of Fourier transform ion cyclotron resonance (FT-ICR) MS/MS and the simultaneous data-independent fragmentation of 2DMS to achieve extensive inter-residue fragmentation of entire proteins. 2DMS was recently developed for top-down proteomics (TDP), and applied to the analysis of calmodulin (CaM), reporting a cleavage coverage of about ~23% using infrared multiphoton dissociation (IRMPD) as fragmentation technique. The goal of this work is to expand the utility of top-down protein analysis using MS/2DMS in order to extend the cleavage coverage in top-down proteomics further into the interior regions of the protein. In this case, using MS/2DMS, the cleavage coverage of CaM increased from ~23% to ~42%.

**Electronic supplementary material:**

The online version of this article (10.1007/s13361-018-1978-y) contains supplementary material, which is available to authorized users.

**Graphical Abstract:**
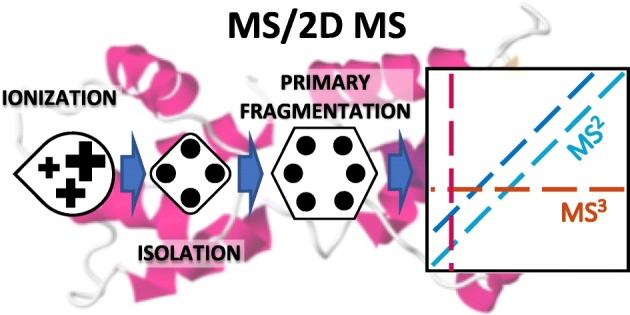
Two-dimensional mass spectrometry, when applied to primary fragment ions from the source, allows deep-sequencing of the protein calmodulin.

## Introduction

The complexity of protein forms has expanded the need for proteomics [1, 2], and technologies such as mass spectrometry (MS) offer a leading platform for characterization of such macromolecules [3]. Studying proteins through MS in their entirety (Top-Down, TDP), rather than from smaller peptides obtained through their enzymatic digestion (Bottom-Up Proteomics, BUP), offers the highest amount of structural information, but constitutes a more challenging experiment often leading to a low sequence coverage of the protein under analysis [4, 5]. The ultra-high resolving power (RP) and mass accuracy of Fourier transform ion cyclotron resonance (FT-ICR) MS [6, 7] alleviate some of the TDP technical limitations, allowing unambiguous characterization of overlapping isotopic distributions and of small mass shifts due to post-translational modifications (PTM) [4, 8].

FT-ICR MS can be used to investigate multiple analytes or charge states in a single two-dimensional (2D) MS experiment, generating a 2D mass spectrum that retains all the MS/MS information from all the precursors in the sample simultaneously [9, 10]. 2DMS was developed in the 1980’s [11–14] but was not pursued after because of computational limitations [15]. With new developments in computer technology and FTMS, the technique was updated with a novel processing software, SPIKE [16], cutting-edge algorithms for the de-noising of 2D mass spectra such as urQRd [17], and computational [18] and tuning optimizations [19, 20]. 2DMS is becoming an efficient analytical tool for the analysis of small molecules [21], macromolecules [22, 23], and proteomics studies [24, 25]. The technique has also been expanded for use in linear ion traps [26] and applications in polymer analysis [27].

**Figure 1:**
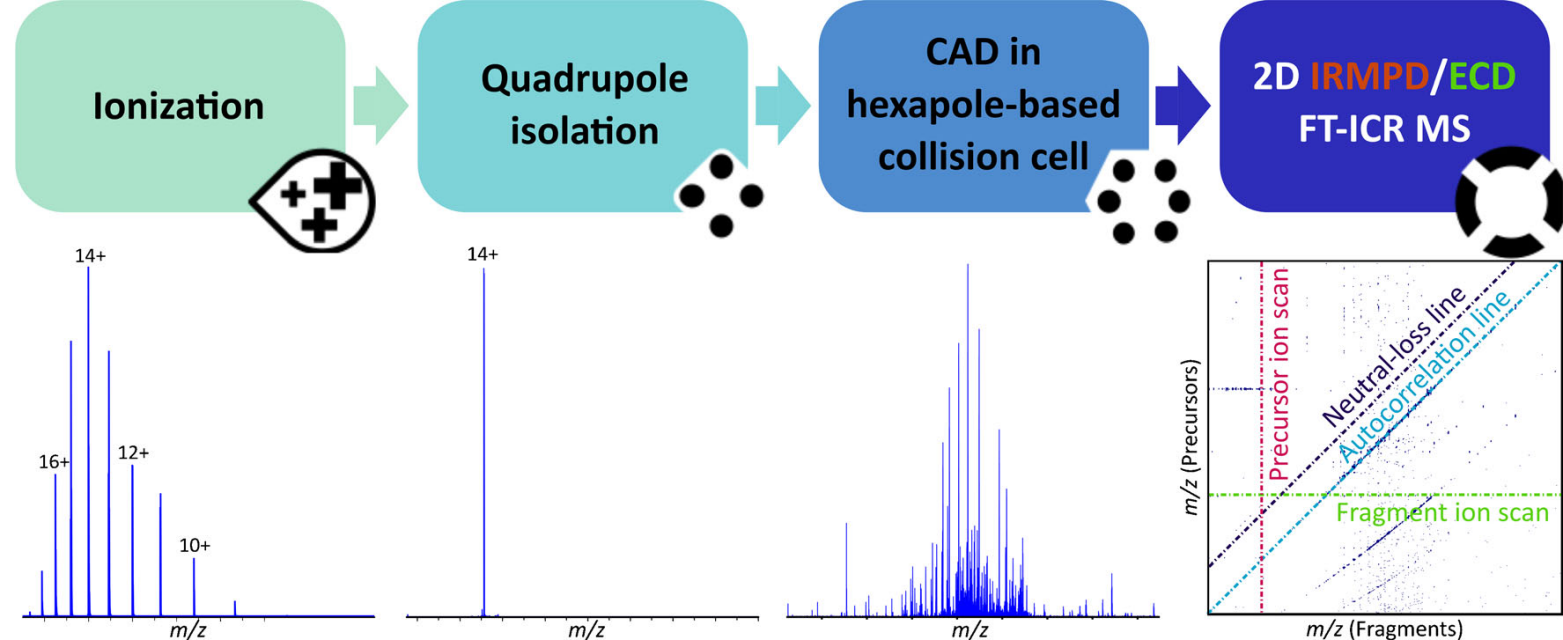
In MS/2DMS, a charge state of interest of the protein under analysis is firstly selected using quadrupolar isolation. The isolated ion species is then fragmented by acceleration into the collision cell using CAD), and the generated CAD-fragments are sent into the ICR-cell for two-dimensional mass spectrometry analysis using IRMPD or ECD as fragmentation techniques. A two-dimensional mass spectrum is generated, containing all the IRMPD/ECD fragmentation patterns of the CAD-fragments, constituting information equivalent to MS^3^ experiments of each and every primary CAD-fragment ion

2DMS has recently been explored for top-down proteomics in a comparative study with 1D MS/MS, using calmodulin (CaM) as a model, and infrared multiphoton dissociation (IRMPD) as fragmentation technique [28]. The study, later implemented with electron-capture dissociation (ECD) [29], showed comparable results between the one-dimensional and two-dimensional analyses, with a considerable saving in time- and sample-consumption for the latter. Although the study demonstrated the suitability of 2DMS for TDP, the reported ~23% cleavage coverage of CaM initiated interest to develop a method for deep sequencing of proteins so as to achieve higher overall inter-residue bond cleavage coverage, particularly into the interior regions of the protein which often show limited fragmentation. This technique is called tandem two-dimensional mass spectrometry (MS/2DMS) [30]. In MS/2DMS a charge state of interest of the protein under analysis is selected through quadrupolar isolation and fragmented in a collision cell (*e.g.* using collisionally-activated dissociation, CAD). Subsequently, the primary fragments are sent to the ICR-cell for simultaneous fragmentation with 2DMS, generating a 2D mass spectrum containing information equivalent to MS^3^ of all primary fragment ions previously generated in the collision cell. The workflow of MS/2DMS is shown in Figure 1. Deeper investigation of macromolecules can be obtained by adding fragmentation steps before introduction of the (fragmented) analytes in the ICR-cell; this further technique is called MS^*n*^/2DMS.

In this work, MS/2DMS is applied to calmodulin to achieve extensive inter-residue fragmentation in a single TDP 2DMS experiment using CAD in the collision cell for the first stage of fragmentation and either ECD or IRMPD for the second stage.

## Experimental

### Materials

Calmodulin from bovine testes was purchased from Sigma-Aldrich (Dorset, UK). HPLC grade methanol and formic acid (HAc), were obtained from Fisher Scientific (Loughborough, UK). Water was purified by a Millipore Direct-Q purification system (Millipore, Nottingham, UK).

CaM (0.4 μM) was dissolved in a 75:25 water/methanol (v/v) solution with 0.3% (v/v) HAc.

### Instrumentation

FT-ICR MS was performed on a 12 T Bruker solariX FT-ICR mass spectrometer (Bruker Daltonik GmbH, Bremen, Germany), using an electrospray ionization (ESI) source. In-source dissociation was used to remove salt adducts. CAD was performed by accelerating the ions to a hexapole collision cell with Argon gas. IRMPD was achieved using a continuous wave, 25 W, CO_2_ laser (Synrad Inc., Washington, USA) held at 70% of its power output. IR photons were produced at a wavelength of 10.6 μm and pulsed into the ICR cell for 0.3-0.5 s prior to detection. ECD was performed generating electrons from a heated hollow cathode (1.5 A) and pulsating them at 10 V into the ICR-cell for 0.2 s prior to detection (ECD bias 1.5-2.0 V).

CAD-MS/IRMPD-2DMS and CAD-MS/ECD-2DMS spectra were acquired respectively with 2048 scans of 512k data-points and 1024 scans of 2M data points over a mass range of *m/z* 368.2-3000 on the vertical axis and *m/z* 147.5-3000 on the horizontal axis; total time of acquisition was ~80 min per experiment. The 2D mass spectra were processed using SPIKE, and de-noised with urQRd (*k* = 20).

See Table S.1 for specific details about the parameters used for the acquisition of the two-dimensional mass spectra and their one-dimensional control spectra.

## Results

**Figure 2:**
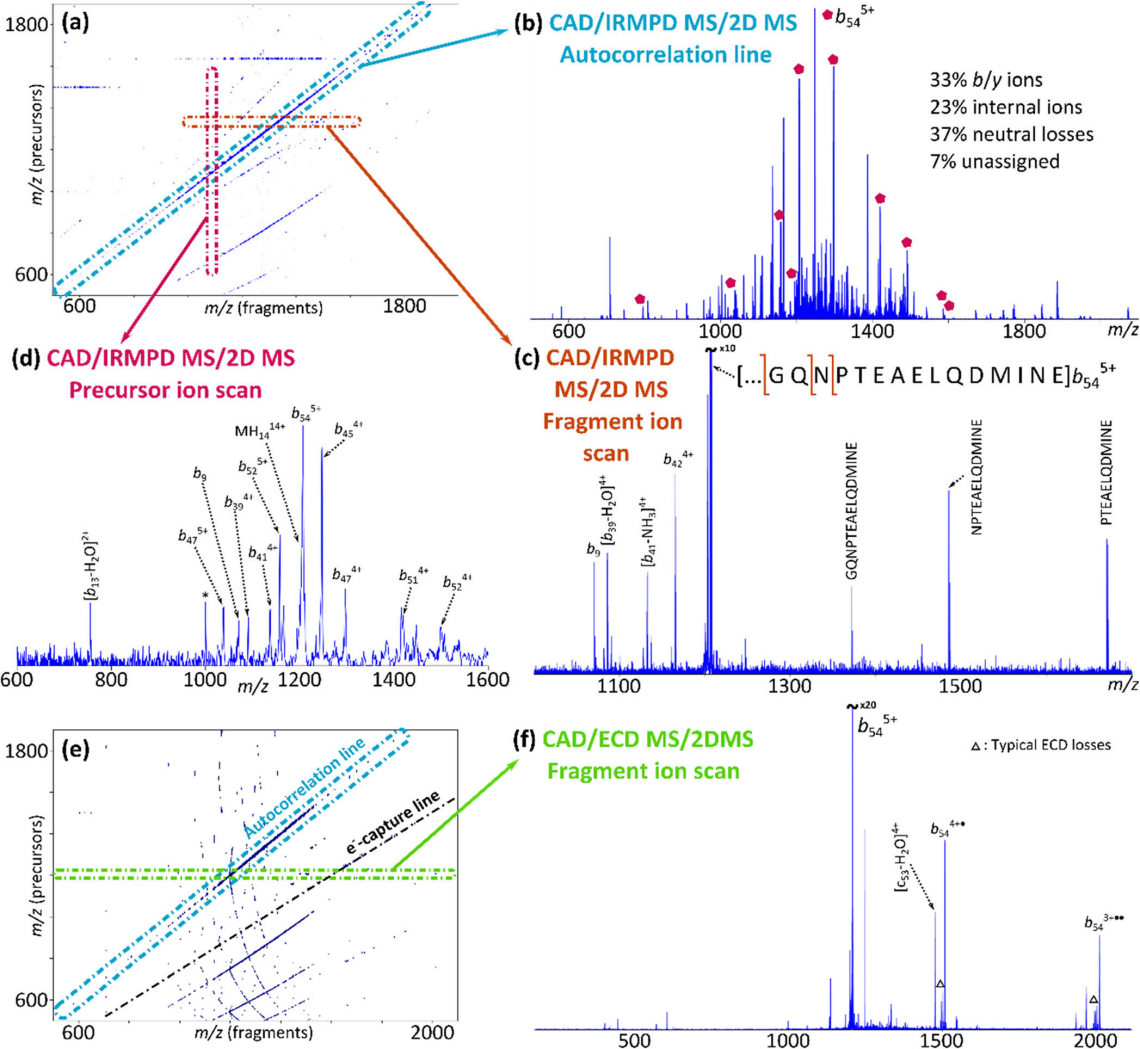
CAD-MS/ECD-2DMS and CAD-MS/IRMPD-2DMS of calmodulin in denaturing conditions. (**a**) Two-dimensional mass spectrum for the CAD-MS/IRMPD-2DMS analysis of CaM. (**b**) Autocorrelation line. (**c**) Fragment ion scan of the CAD-ion *b*_*54*_^*5+*^. (**d**) Vertical ion scan for the ion *b*_*9*_. The assigned ions are labelled on the autocorrelation line (spectrum b) with a pentagon. (**e**) 2D mass spectrum from the analysis of CaM with CAD-MS/ECD-2DMS. (**f**) Fragment ion scan of the ion *b*_*54*_^*5+*^

Figure 2 shows the results of the CAD-MS/IRMPD-2DMS and CAD-MS/ECD-2DMS analyses of CaM. The resulting 2D mass spectra are reported respectively in figures 2.a and 2.e.

The autocorrelation line is extracted from the CAD-MS/IRMPD-2DMS mass spectrum and reported in Figure 2.b, showing a large number of *b*/*y* ions, neutral losses, and internal fragments (reported in percentage): typical ion fragments generated by techniques such as CAD.

Figure 2.c shows the extraction of a horizontal ion scan, corresponding to the fragmentation pattern of the CAD fragment *b*_54_^5+^ (*m/z* 1206.3786), generated during the fragmentation period inside the ICR-cell. Many *b* and internal ions and neutral losses are recognized in the spectrum, as expected from IRMPD. The horizontal scan of ion *b*_54_^5+^ was calibrated using the theoretical *m/z* of the recognized fragments, and the obtained fitting parameters were used to calibrate the entire two-dimensional mass spectrum with a quadratic equation. Fragment ions could be assigned with a mass accuracy of 0.21±0.98 ppm. Horizontal fragment ion scans were extracted for all the successfully assigned precursors, and their fragmentation patterns assigned and used to calculate the cleavage coverage of CaM with the CAD-MS/IRMPD-2DMS MS/2DMS experiment, corresponding to ~41%.

A vertical scan is extracted and shown in Figure 2.d. This mass spectrum shows all the precursors that generate the ion *b*_*9*_ (*m/z* 1070.5000) during the fragmentation period in the ICR-cell. Figure 2.d shows about 15 precursors, which can only be N-terminal precursor ions and the molecular ion MH_14_^14+^ of CaM. Precursor ions were assigned through cross-correlation with the autocorrelation line. Signals due to experimental noise are labelled with a star symbol (*). The *b* ions assigned through the extraction of the vertical ion scan are labelled in Figure 2.b with a pentagon.

A similar analysis has been performed for the spectrum of Figure 2.e, and it is detailed in the Supporting Information (see also Table S.3 and Figure S.3). The cleavage coverage for the CAD-MS/ECD-2DMS experiment is ~33%

Combination of the data obtained with the CAD-MS/IRMPD-2DMS and CAD-MS/ECD-2DMS MS/2DMS experiments of CaM led to a cumulative cleavage coverage of ~42%.

## Discussion

The tandem two-dimensional mass spectrometric analysis of CaM generated two 2D mass spectra retaining information equivalent to MS^3^ using CAD as primary fragmentation and IRMPD or ECD as secondary fragmentation techniques.

**Figure 3:**
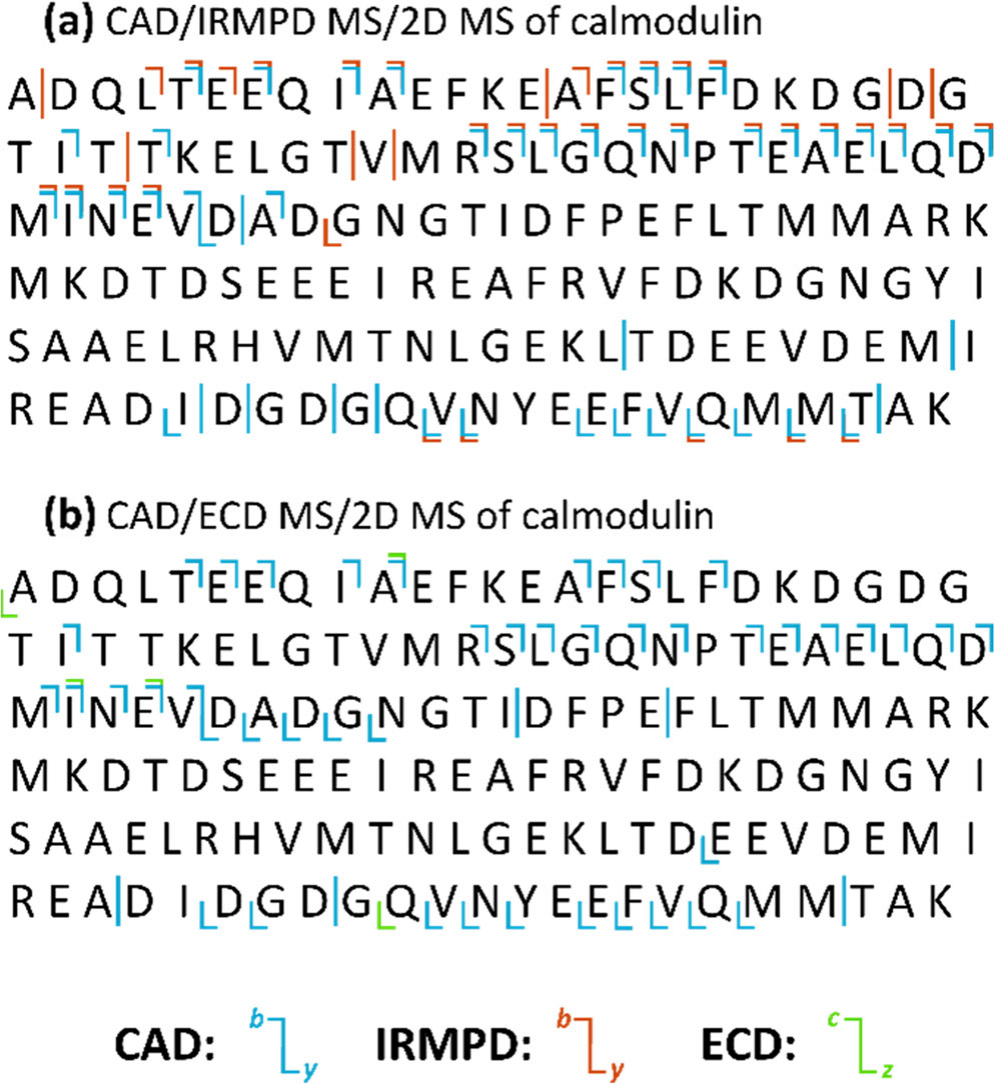
Cleavage coverage maps for the CAD-MS/IRMPD-2DMS and CAD-MS/ECD-2DMS analysis of CaM in denaturing conditions. Vertical lines indicate cleavages from internal fragments. The total cleavage coverage for the MS/2DMS analysis of CaM is ~42%

Interpretation of these spectra relies on the analysis of the autocorrelation line, bearing information equivalent to CAD MS/MS, and the extraction of a horizontal line for each primary fragment, showing their fragmentation patterns and constituting information equivalent to MS^3^. A cleavage coverage map has been built for each analysis, reporting in blue the fragments assigned to the autocorrelation line, and in orange (IRMPD) and green (ECD) the fragments assigned to the horizontal ion scans (Figure 3). Similar to what is reported for the TDP 2D IRMPD MS studies of CaM, the cleavage sites are prevalent in proximity of the protein termini. Such phenomenon is due to the similarity of IRMPD and CAD, used here as primary fragmentation, as ergodic processes. In MS/2DMS the primary fragments are further fragmented using IRMPD or ECD, allowing more detailed characterization of the protein. However, if no primary fragments are generated from the most internal parts of the protein, further fragmentation will not cover missing cleavages. A method to improve the cleavage coverage on this purpose would involve protein unfolding (*e.g.* through supercharging), or the use of a “non-ergodic” fragmentation such as electron-transfer dissociation before the ICR-cell. It is important to notice that, in order to obtain extensive secondary fragmentations, the fragmentation efficiency of the primary fragmentation should be high. A dissociation technique with high fragmentation efficiency will generate abundant primary fragment ions, which can be further fragmented in the ICR-cell.

Investigation of the horizontal scans represents the equivalent of adding MS^3^ information to the MS^2^ information provided by the analysis of the autocorrelation line. The cleavage coverage map of Figure 3.a shows in red the MS^3^ information obtained with IRMPD as secondary fragmentation technique. Many cleavages occur in the same sites as the primary CAD fragmentation (due to the similarity of the dissociation techniques). However, further fragmentation with IRMPD increased the cleavage coverage of the protein by ~9%. On the other hand, ECD did not produce extensive information. It is hypothesized that such result is due to the low fragmentation efficiency of ECD compared to IRMPD. Standard 1D ECD FT-ICR MS/MS experiments require a high number of transient accumulations (with an increase of signal-to-noise ratio, S/N, proportional to the square root of the number of scans) in order to visualize fragments over the limits of detection. In 2DMS, transients cannot be accumulated to improve S/N in the horizontal dimension, but every iteration of the pulse programme used to obtain the precursor/fragment correlation (scans in *t*_1_) increases the vertical resolution. Finally, ECD generally requires a high precursor ion abundance to produce high-quality tandem mass spectra. Optimization of the fragmentation efficiency of the primary dissociation and/or better storage of the primary fragments in the ICR-cell are relevant parameters on this purpose. Analysis of the horizontal ion scans extracted from the CAD-MS/ECD-2DMS spectrum increased the cleavage coverage of the protein by ~1% (Figure 5.b). Further studies are in progress to increase the efficiency of ECD in TDP 2DMS.

Further information about the protein structure (and dissociation mechanisms) can be obtained by extracting the vertical (precursor) ion scans. Extracting a vertical ion scan from a 2D mass spectrum produces a spectrum of all the precursors that generated the fragment of interest. In MS/2DMS precursor ions are primary fragments, *i.e.* fragments that retain a protein terminus (C- or N-), or internal fragments. Fragment ions that retain a terminus (mainly *b/y* in case of IRMPD, or *c/z* for ECD) can be generated only by a precursor with the same terminus. This is particularly important for *de novo* sequencing with MS/2DMS, for which, once a secondary fragment ion has been identified, extraction of its vertical ion scan will show precursors that can have the same terminus. In MS/2DMS precursor ion scans have the potential to easily discriminate primary fragments based on their termini. Figure 2.d shows an example with the secondary IRMPD ion *b*_9_, whose precursors can only be other *b* ions (or eventually any higher *a* ion, generated by a secondary fragmentation path of CAD) and the remaining unfragmented molecular ion. Vertical ion scans are a useful feature of 2DMS, but their resolution is often much lower compared to the horizontal dimension because of their proportional dependence to the number of experimental scans, hence to the experimental time [28]. Recent developments in data acquisition for 2DMS allow improvement of the vertical resolution without impairing acquisition times, although increasing processing times [18].

Notably, MS/2DMS experiments have a longer acquisition time compared to standard 2DMS: ~80 min compared to ~20 min, because in TDP, primary fragment ions will overlap heavily in m/z. Thus, the required vertical resolution, which is higher for MS/2DMS compared to TDP 2DMS.

As the technique in the early stages of development, MS/2DMS does not yet provide cleavage coverage comparable with other studies about deep sequencing of proteins that used similar combinations of techniques [31], or other fragmentation techniques, such as ultraviolet photodissociation (UVPD) [32]. Nonetheless, the technique has unrealized potential. An as yet unexplored configuration for TDP MS/2DMS analysis includes the use of ETD as the primary fragmentation, and laser/electron-based activations as the secondary fragmentations. In addition to the more “random” nature of low-energy electron-based fragmentation techniques, which allow them to cleave deeper in the protein, their “non-ergodic” mechanisms allow fragmentation with the possibility of leaving intact the most labile PTM’s, which would be lost with CAD. It is hypothesized here that use of ETD-MS/(IRMPD or UVPD)-2DMS would improve fragmentation and sequence coverage of larger macromolecules, and in particular for post-translationally modified proteins. Another interesting configuration involves the use of ETD-MS/ECD-2DMS, and the respective formation of charge-reduced radical species and fragments, thus generating extensive in-cell electron-based fragmentation. There are quite a few MS^n^/2DMS configurations yet to explore.

Finally, although MS/2DMS still has room for improvement, its use in the TDP analysis of CaM raised the cleavage coverage compared to 2D IRMPD MS. This study represents a further step in the analysis of CaM by two-dimensional mass spectrometry. Including previously published BUP and TDP 2DMS data [28, 29], and the MS/2DMS data herein, the aggregate, cumulative cleavage coverage of calmodulin is now ~76%.

## Conclusions

MS/2DMS is a promising tool for deep sequencing of proteins, providing a new fragmentation tool for top-down proteomics. In this work, the cleavage coverage of calmodulin increased from ~23% to ~42% compared to the top-down 2DMS of the protein alone.

## Electronic supplementary material

ESM 1 (DOCX 248 kb)
