# Supplementary material for: Application of Tandem Two-Dimensional Mass Spectrometry for Top-Down Deep Sequencing of Calmodulin
Source: J Am Soc Mass Spectrom. 2018 Jun 4;29(8):1700–5. doi: 10.1007/s13361-018-1978-y (PMC6060996; doi:10.1007/s13361-018-1978-y)
Supplement: Supplementary file 1 — (DOCX 248 kb) [file 13361_2018_1978_MOESM1_ESM.docx]

# Supporting Information – Application of tandem two-dimensional mass spectrometry for Top-Down deep sequencing of Calmodulin

Federico Floris,^a^ Lionel Chiron,^b^ Alice M. Lynch,^a^ Mark P. Barrow,^a^ Marc-André Delsuc,^b,c^ Peter B. O’Connor.^a,1^

^a^University of Warwick, Department of Chemistry, Coventry, CV4 7AL, United Kingdom

^b^CASC4DE, 20 Avenue du Neuhof, 67100, Strasbourg, France

^c^Institut de Génétique et de Biologie Moléculaire et Cellulaire, Institut National de la Santé et de la Recherche, U596; Centre National de la Recherche Scientifique, Unité Mixte de Recherche 7104; Université de Strasbourg, 67404 Illkirch-Graffenstaden, France

^1^Corresponding Author: Peter B. O’Connor, University of Warwick, Department of Chemistry, Coventry, CV4 7AL, United Kingdom. Email: [p.oconnor@warwick.ac.uk](mailto:p.oconnor@warwick.ac.uk). Tel: +44 (0) 2476 151 008.

Keywords: FT-ICR MS, 2D MS, Top-down proteomics

Contents

[Supporting Information – Application of tandem two-dimensional mass spectrometry for Top-Down deep sequencing of Calmodulin 1](#_Toc505191328)

[List of figures 1](#_Toc505191329)

[List of tables 1](#_Toc505191330)

[Mass Spectrometry of Calmodulin 2](#_Toc505191331)

[One-dimensional MS of Calmodulin 2](#_Toc505191332)

[MS/2D MS of Calmodulin 8](#_Toc505191333)

[Parameters for MS acquisition of Calmodulin 16](#_Toc505191334)

## List of figures

Figure S.1: Full mass spectrum of calmodulin, showing the range of charge states reached by the protein in denaturing conditions. Transients have been accumulated for 8 scans. 2

Figure S.2: (a) CAD MS/MS spectrum of calmodulin. It has been obtained isolating in the quadrupole of the mass spectrometer the 14+ charge state of calmodulin and accelerating the ions into Argon in a hexapole-based collision cell. Mass analysis and detection were performed in the ICR-cell. (b) Cleavage coverage map of the spectrum reported in (a): the cleavage coverage reaches 37.8% considering the generated b/y ions, and raises to 42.1% if internal ions are added. Complete mass assignments for the showed spectrum and coverage map are shown in table S.1. 3

Figure S.3: Autocorrelation line extracted from the CAD/ECD MS/2D MS spectrum of CaM. 16

## List of tables

[Table S.1: Fragments assigned to the one-dimensional CAD MS/MS spectrum of Calmodulin in denaturing conditions. 3](#_Toc505191337)

[Table S.2: CAD fragments assigned to the autocorrelation line of the CAD/IRMPD MS/2D MS experiment on CaM. 9](#_Toc505191338)

[Table S.3: CAD fragments assigned to the autocorrelation line of the CAD/ECD MS/2D MS experiment on CaM. 12](#_Toc505191339)

[Table S.4: Parameters used for the acquisition of the one-dimensional and two-dimensional mass spectra of Calmodulin. 16](#_Toc505191340)

## Mass Spectrometry of Calmodulin

### One-dimensional MS of Calmodulin

#### Full MS of CaM

A one-dimensional full mass spectrum of Calmodulin (CaM, 17kDa) in denaturing conditions was acquired as control. The spectrum was acquired using 4M data-points (16 bits), for a transient length of 1.6777 s, and a resulting resolving power of ~340000. Calibration was achieved through instrument calibration with a quadratic curve, using an external calibrant before the data acquisition. The full mass spectrum of CaM in denaturing conditions is shown in Figure S.1. It exhibits a wide range of charge states reached by calmodulin at the detailed conditions, from 8+ to 18+. The most abundant charge state, corresponding to MH_14_^14+^ (*m/z* 1199.5681), was isolated and accumulated in the quadrupole, for MS/MS fragmentation with CAD in the hexapole collision cell.


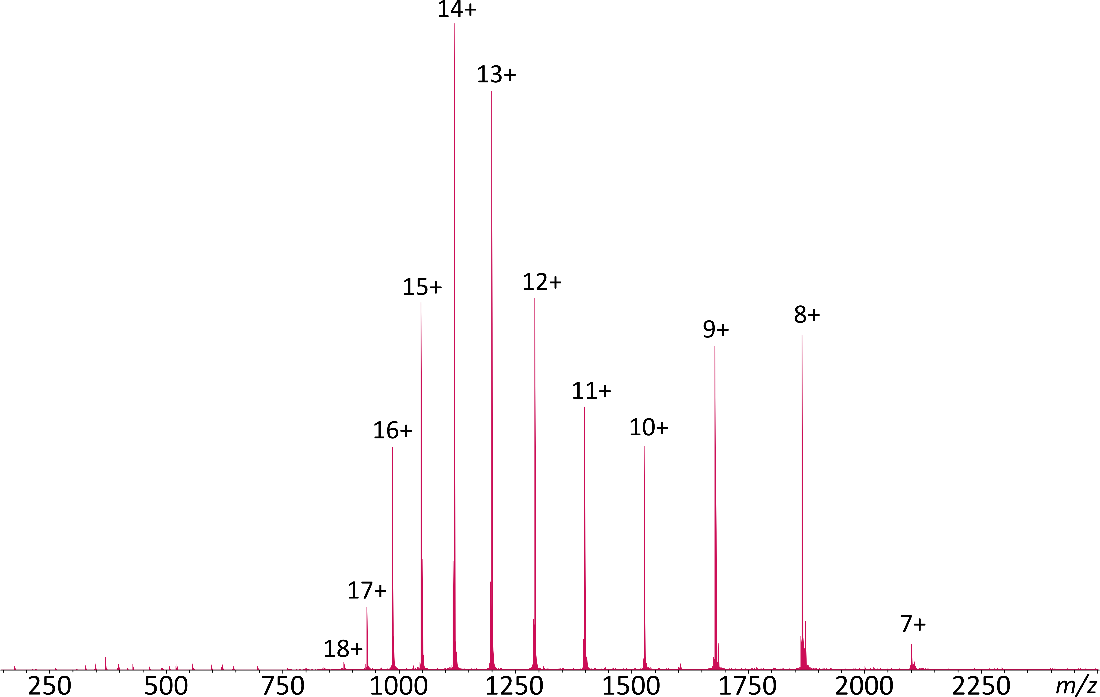


Figure S.1: Full mass spectrum of calmodulin, showing the range of charge states reached by the protein in denaturing conditions. Transients have been accumulated for 8 scans.

#### CAD MS/MS of calmodulin

A one-dimensional CAD MS/MS spectrum for CaM was acquired with the same acquisition parameters listed for the full MS (Figure S.2.a). The resulting resolving power was ~340000. The mass spectrum was internally calibrated using theoretical mass-to-charge ratios of the recognized peaks. CAD of calmodulin exhibited a large number of multiply charged *b*/*y* ions (the vast majority represented by *b* ions) matched by neutral losses in large scale. It is hypothesized that the unassigned peaks are originated by “sequence scramble”, typical of *b* ions in MS/MS experiments involving techniques such as CAD and IRMPD. Ions could be assigned with a high average mass accuracy of 0.21±0.98 ppm, leading to a cleavage coverage of 37.8% considering only *b* and *y* ions, and 41.2% including the assigned internal ions. A complete list of the assigned fragments for the 1D CAD MS/MS spectrum of CaM in denaturing conditions is reported in the Table S.28, along with the respective cleavage coverage map (Figure S.2.b).


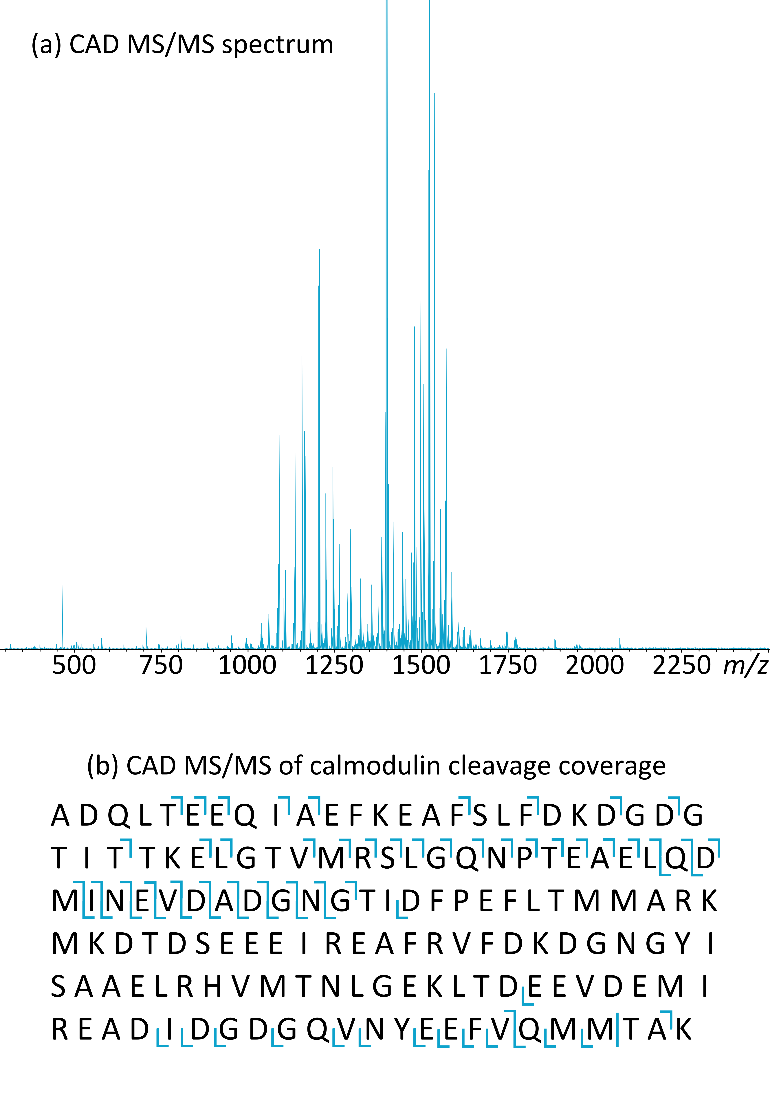


Figure S.2: (a) CAD MS/MS spectrum of calmodulin. It has been obtained isolating in the quadrupole of the mass spectrometer the 14+ charge state of calmodulin and accelerating the ions into Argon in a hexapole-based collision cell. Mass analysis and detection were performed in the ICR-cell. (b) Cleavage coverage map of the spectrum reported in (a): the cleavage coverage reaches 37.8% considering the generated b/y ions, and raises to 42.1% if internal ions are added. Complete mass assignments for the showed spectrum and coverage map are shown in table S.1.

Table S.1: Fragments assigned to the one-dimensional CAD MS/MS spectrum of Calmodulin in denaturing conditions.

| **Ion** | **Charge** | **Observed m/z** | **Theoretical m/z** | **Error [ppm]** |
| --- | --- | --- | --- | --- |
| y4 | 1 | 450.237927 | 450.238082 | -0.34 |
| AELQD | 1 | 557.256468 | 557.256570 | -0.18 |
| b5 | 1 | 571.272036 | 571.272220 | -0.32 |
| y5 | 1 | 581.278567 | 581.278567 | 0.00 |
| DMINE | 1 | 603.244246 | 603.244291 | -0.07 |
| NPTEAE | 1 | 642.272905 | 642.272949 | -0.07 |
| NEVDAD/ADIDGDG | 1 | 644.252191 | 644.252214 | -0.04 |
| b6-H2O | 1 | 682.304283 | 682.304249 | 0.05 |
| y6-NH3 | 1 | 692.310630 | 692.310596 | 0.05 |
| ? | 1 | 702.313161 |  |  |
| y6 | 1 | 709.337144 | 709.337145 | 0.00 |
| y12 | 2 | 745.836430 | 745.836347 | 0.11 |
| PTEAELQ | 1 | 769.372692 | 769.372663 | 0.04 |
| y13 | 2 | 795.370767 | 795.370554 | 0.27 |
| AELQDMI | 1 | 801.381432 | 801.381119 | 0.39 |
| y7 | 1 | 808.405852 | 808.405559 | 0.36 |
| EAELQDM/DMINEVD | 1 | 817.339856 | 817.339649 | 0.25 |
| b7 | 1 | 829.357023 | 829.357408 | -0.46 |
| ? | 2 | 835.894444 |  |  |
| LQDMINE/ELQDMIN | 1 | 844.387118 | 844.386933 | 0.22 |
| QNPTEAEL/NPTEAELQ | 1 | 883.415619 | 883.415591 | 0.03 |
| ? | 1 | 884.400036 |  |  |
| AELQDMIN | 1 | 915.424053 | 915.424047 | 0.01 |
| LQDMINEV | 1 | 943.455433 | 943.455347 | 0.09 |
| b16 | 2 | 946.949345 | 946.949267 | 0.08 |
| y8 | 1 | 955.474257 | 955.473973 | 0.30 |
| b44-NH3 | 5 | 968.476188 | 968.472885 | 3.41 |
| ELQDMINE | 1 | 973.429364 | 973.429527 | -0.17 |
| y17 | 2 | 973.934603 | 973.934779 | -0.18 |
| b45-H2O | 5 | 994.084640 | 994.084600 | 0.04 |
| b45 | 5 | 997.688673 | 997.686713 | 1.96 |
| NPTEAELQD | 1 | 998.442806 | 998.442535 | 0.27 |
| DMINEVDAD | 1 | 1003.403477 | 1003.403707 | -0.23 |
| b27-H2O | 3 | 1008.477013 | 1008.476739 | 0.27 |
| b46 | 5 | 1011.894279 | 1011.894136 | 0.14 |
| ? | 2 | 1013.977790 |  |  |
| ? | 2 | 1014.976509 |  |  |
| ? | 1 | 1015.439915 |  |  |
| y18 | 2 | 1031.448051 | 1031.448251 | -0.19 |
| b47-H2O | 5 | 1034.100078 | 1034.100542 | -0.45 |
| b37-H2O | 4 | 1035.758243 | 1035.758406 | -0.16 |
| b47 | 5 | 1037.702782 | 1037.702655 | 0.12 |
| ? | 2 | 1039.005216 |  |  |
| b37 | 4 | 1040.261179 | 1040.261048 | 0.13 |
| AELQDMINE/EAELQDMIN | 1 | 1044.466910 | 1044.466641 | 0.26 |
| b28 | 3 | 1048.163534 | 1048.162820 | 0.68 |
| b48-H2O | 5 | 1056.718425 | 1056.717355 | 1.01 |
| b38-H2O | 4 | 1057.514582 | 1057.516414 | -1.73 |
| b48 | 5 | 1060.319520 | 1060.319468 | 0.05 |
| b38 | 4 | 1062.019406 | 1062.019055 | 0.33 |
| b9 | 1 | 1070.499793 | 1070.500050 | -0.24 |
| b29-H2O | 3 | 1075.842010 | 1075.841858 | 0.14 |
| y19-H2O | 2 | 1078.997600 | 1078.985001 |  |
| b39-2H2O | 4 | 1081.285086 | 1081.284788 | 0.28 |
| b49-NH3 | 5 | 1082.524153 | 1082.525873 | -1.59 |
| y9 | 1 | 1084.516569 | 1084.516567 | 0.00 |
| b39-H2O | 4 | 1085.787276 | 1085.787430 | -0.14 |
| b49 | 5 | 1085.931256 | 1085.931183 | 0.07 |
| y19 | 2 | 1087.990234 | 1087.990283 | -0.05 |
| b39 | 4 | 1090.289860 | 1090.290071 | -0.19 |
| b40-H2O | 4 | 1100.042667 | 1100.042796 | -0.12 |
| b40 | 4 | 1104.545595 | 1104.545437 | 0.14 |
| b50-H2O | 5 | 1105.335035 | 1105.334459 | 0.52 |
| b50 | 5 | 1108.936466 | 1108.936572 | -0.10 |
| ? | 5 | 1119.945778 |  |  |
| b19 | 2 | 1120.547587 | 1120.541520 | 5.41 |
| ? | 2 | 1121.026046 |  |  |
| b51-H2O | 5 | 1131.543610 | 1131.542556 | 0.93 |
| b41-H2O | 4 | 1132.057416 | 1132.057440 | -0.02 |
| b51 | 5 | 1135.144437 | 1135.144669 | -0.20 |
| b41 | 4 | 1136.559763 | 1136.560081 | -0.28 |
| ? | 4 | 1140.064673 |  |  |
| b10 | 1 | 1141.537034 | 1141.537164 | -0.11 |
| GEuLTDEEV ? | 1 | 1143.534992 |  |  |
| y50-2H2O | 5 | 1148.750259 | 1148.745055 | 4.53 |
| ? | 5 | 1152.162462 |  |  |
| b52-H2O | 5 | 1154.159419 | 1154.159369 | 0.04 |
| b42-2H2O | 4 | 1156.060163 | 1156.065531 | -4.64 |
| b52 | 5 | 1157.761029 | 1157.761482 | -0.39 |
| b42-H2O | 4 | 1160.568790 | 1160.568172 | 0.53 |
| b42-H2O deamidated? | 4 | 1160.812829 | 1160.814176 | -1.16 |
| y30 | 3 | 1164.514272 | 1164.514431 | -0.14 |
| b42 | 4 | 1165.070668 | 1165.070813 | -0.12 |
| b31 | 3 | 1167.557724 | 1167.557899 | -0.15 |
| ? | 5 | 1168.347567 |  |  |
| NYEEFVQMM | 1 | 1172.475149 | 1172.475097 | 0.04 |
| ? | 3 | 1173.173708 |  |  |
| b53-H2O | 5 | 1176.966852 | 1176.967954 | -0.94 |
| b53 | 5 | 1180.569687 | 1180.570067 | -0.32 |
| GQNPTEAELQD | 1 | 1183.522644 | 1183.522577 | 0.06 |
| ? | 1 | 1186.545941 |  |  |
| ELQDMINEVD | 1 | 1187.524749 | 1187.524885 | -0.11 |
| b43 | 4 | 1189.333917 | 1189.334004 | -0.07 |
| b54-H2O | 5 | 1202.776165 | 1202.776473 | -0.26 |
| b32 | 3 | 1205.252069 | 1205.252587 | -0.43 |
| b54 | 5 | 1206.377652 | 1206.378586 | -0.77 |
| ? | 5 | 1209.382380 |  |  |
| ? | 4 | 1210.344746 |  |  |
| y10 | 1 | 1213.558961 | 1213.559161 | -0.16 |
| b44 | 4 | 1214.595986 | 1214.595924 | 0.05 |
| ? | 5 | 1219.181231 |  |  |
| ? | 5 | 1220.586816 |  |  |
| b55-H2O | 5 | 1222.587979 | 1222.590156 | -1.78 |
| b55 | 5 | 1226.191471 | 1226.192269 | -0.65 |
| b45-H2O | 4 | 1242.353350 | 1242.353931 | -0.47 |
| PTEAELQDMIN/NPTEAELQDMI | 1 | 1242.566821 | 1242.567084 | -0.21 |
| b56-H2O | 5 | 1245.597006 | 1245.595545 | 1.17 |
| b45 | 4 | 1246.855858 | 1246.856573 | -0.57 |
| b56 | 5 | 1249.197215 | 1249.197658 | -0.35 |
| b34-H2O | 3 | 1251.937639 | 1251.938780 | -0.91 |
| ? | 5 | 1256.401097 |  |  |
| ? | 5 | 1258.005198 |  |  |
| AELQDMINEVD/ELQDMINEVDA | 1 | 1258.562157 | 1258.561999 | 0.13 |
| b57-H2O | 5 | 1259.802040 | 1259.802968 | -0.74 |
| b46-H2O | 4 | 1260.112713 | 1260.113210 | -0.39 |
| b57 | 5 | 1263.404712 | 1263.405081 | -0.29 |
| b46 | 4 | 1264.617946 | 1264.615851 | 1.66 |
| b58-H2O | 5 | 1282.806987 | 1282.808356 | -1.07 |
| b35-H2O | 3 | 1284.961439 | 1284.961584 | -0.11 |
| b58 | 5 | 1286.409909 | 1286.410469 | -0.44 |
| b35 | 3 | 1290.964525 | 1290.965106 | -0.45 |
| b47-H2O | 4 | 1292.373099 | 1292.373858 | -0.59 |
| b59-NH3 | 5 | 1294.413142 |  |  |
| b47 | 4 | 1296.876108 | 1296.876500 | -0.30 |
| b59 | 5 | 1297.814850 | 1297.814762 | 0.07 |
| b22 | 2 | 1299.616655 | 1299.615946 | 0.55 |
| y91 | 8 | 1309.489188 | 1309.490072 | -0.68 |
| y92 | 8 | 1318.369091 | 1318.369711 | -0.47 |
| b60 | 5 | 1320.620332 | 1320.623348 | -2.28 |
| b48-H2O | 4 | 1320.645719 | 1320.644874 | 0.64 |
| b48 | 4 | 1325.146922 | 1325.147516 | -0.45 |
| b61 | 5 | 1332.026345 | 1332.027641 | -0.97 |
| b36 | 3 | 1334.645760 | 1334.645268 | 0.37 |
| y94-H2O | 8 | 1342.879079 | 1342.880310 | -0.92 |
| y94 | 8 | 1345.127751 | 1345.131631 | -2.88 |
| ? | 6 | 1346.299331 |  |  |
| b49-H2O | 4 | 1352.659724 | 1352.659519 | 0.15 |
| NPTEAELQDMIN | 1 | 1356.610107 | 1356.610012 | 0.07 |
| b49 | 4 | 1357.161036 | 1357.162160 | -0.83 |
| y95 | 8 | 1361.260171 | 1361.261955 | -1.31 |
| PTEAELQDMINE | 1 | 1371.609391 | 1371.609678 | -0.21 |
| y96 | 8 | 1375.515908 | 1375.517321 | -1.03 |
| b24 | 2 | 1385.640148 | 1385.640150 | 0.00 |
| b50 | 4 | 1385.918432 | 1385.918896 | -0.33 |
| y97-NH3 | 8 | 1387.523731 | 1387.524511 | -0.56 |
| y97 | 8 | 1389.652007 | 1389.652829 | -0.59 |
| MH12 | 12 | 1399.491419 | 1399.328225 |  |
| b51-H20 | 4 | 1414.176371 | 1414.176376 | 0.00 |
| y85 | 7 | 1416.810961 | 1416.809822 | 0.80 |
| b51 | 4 | 1418.678368 | 1418.679017 | -0.46 |
| y99 | 8 | 1420.409916 | 1420.411258 | -0.94 |
| b52-H2O | 4 | 1442.445725 | 1442.447392 | -1.16 |
| AELQDMINEVDAD | 1 | 1444.626205 | 1444.626057 | 0.10 |
| b52 | 4 | 1446.948579 | 1446.950033 | -1.01 |
| y88 | 7 | 1455.544814 | 1455.545995 | -0.81 |
| b142 ? | 11 | 1462.053385 | 1462.054687 | -0.89 |
| PTEAELQDMINEV | 1 | 1470.676098 | 1470.678092 | -1.36 |
| ? | 5 | 1471.701777 |  |  |
| y89 | 7 | 1471.836657 | 1471.837842 | -0.80 |
| ? | 10 | 1473.204367 |  |  |
| b53 | 4 | 1475.459454 | 1475.460765 | -0.89 |
| y90 | 7 | 1479.981875 | 1479.983765 | -1.28 |
| NPTEAELQDMINE | 1 | 1485.652314 | 1485.652606 | -0.20 |
| y12 | 1 | 1490.665076 | 1490.665418 | -0.23 |
| y91 | 7 | 1496.414728 | 1496.416186 | -0.97 |
| y92 | 7 | 1506.561549 | 1506.564345 | -1.86 |
| b54 | 4 | 1507.722469 | 1507.721414 | 0.70 |
| ? | 11 | 1511.526555 |  |  |
| ? | 11 | 1513.163836 |  |  |
| y93 | 7 | 1522.994678 | 1522.996765 | -1.37 |
| b55 | 4 | 1532.487252 | 1532.488517 | -0.83 |
| ? | 7 | 1534.431923 |  |  |
| y94 | 7 | 1537.149174 | 1537.149396 | -0.14 |
| ? | 7 | 1539.437860 |  |  |
| y95 | 7 | 1555.583270 | 1555.584052 | -0.50 |
| b56 | 4 | 1561.244756 | 1561.245253 | -0.32 |
| ? | 7 | 1569.300076 |  |  |
| y96 | 7 | 1571.874581 | 1571.875899 | -0.84 |
| b57 | 4 | 1579.004183 | 1579.004532 | -0.22 |
| NPTEAELQDMINEV | 1 | 1584.720438 | 1584.721020 | -0.37 |
| ? | 7 | 1585.598201 |  |  |
| y97 | 7 | 1588.031568 | 1588.030765 | 0.51 |
| ? | 7 | 1588.141701 |  |  |
| ? | 7 | 1604.461720 |  |  |
| ? | 7 | 1604.715737 |  |  |
| y98 | 7 | 1606.747812 | 1606.750835 | -1.88 |
| b59-3H2O | 4 | 1608.008575 | 1608.508710 |  |
| QNPTEAELQDMINE | 1 | 1613.709959 | 1613.711184 | -0.76 |
| y99-NH3 ? | 7 | 1620.895391 | 1620.750891 |  |
| b59 | 4 | 1622.017828 | 1622.016634 | 0.74 |
| y99 | 7 | 1623.184649 | 1623.183255 | 0.86 |
| y100-NH3 | 7 | 1639.188791 | 1639.044974 |  |
| y100 | 7 | 1641.475669 | 1641.477338 | -1.02 |
| b60 | 4 | 1650.527695 | 1650.527366 | 0.20 |
| PTEAELQDMINEVDA | 1 | 1656.740351 | 1656.742150 | -1.09 |
| GQNPTEAELQDMINE | 1 | 1670.732911 | 1670.732648 | 0.16 |
| ? | 1 | 1682.720795 |  |  |
| QNPTEAELQDMINEV | 1 | 1712.777743 | 1712.779598 | -1.08 |
| ? | 3 | 1735.165029 |  |  |
| DGDGQVNYEEFVQMM | 1 | 1743.699284 | 1743.698905 | 0.22 |
| ? | 2 | 1746.266922 |  |  |
| GQNPTEAELQDMINEV | 1 | 1769.800627 | 1769.801062 | -0.25 |
| PTEAELQDMINEVDAD | 1 | 1771.768377 | 1771.769094 | -0.40 |
| y15 | 1 | 1774.812276 | 1774.813874 | -0.90 |
| QNPTEAELQDMINEVD | 1 | 1827.804265 | 1827.806542 | -1.25 |
| ? | 1 | 1867.800147 |  |  |
| GQNPTEAELQDMINEVD | 1 | 1884.828461 | 1884.828006 | 0.24 |
| NPTEAELQDMINEVDADG/PTEAELQDMINEVDADGN | 1 | 1942.830820 | 1942.833486 | -1.37 |
| y17 | 1 | 1946.862280 | 1946.862282 | 0.00 |
| GQNPTEAELQDMINEVDA | 1 | 1955.863039 | 1955.865120 | -1.06 |
| QNPTEAELQDMINEVDADG/GQNPTEAELQDMINEVDAD | 1 | 2070.890318 | 2070.892064 | -0.84 |
|  |  | Average Error [ppm] | | -0.21 |
|  |  | STD | | 0.98 |

### MS/2D MS of Calmodulin

#### CAD/IRMPD MS/2D MS of CaM

The 2D spectrum of Figure 2.a was acquired with 2k data-points in the vertical dimension and 512k data-points (16 bit) in the horizontal dimension. The vertical resolving power was RP_v_ ≈ 1500, while the horizontal resolving power was RP_h_ ≈ 60000, both reported at *m/z* 400. The resulting total resolving power for the 2D mass spectrum of Figure 2.a is RP_2D_ ≈ 90000000, reported at *m/z* 400. The instrument was externally calibrated using a standard known mixture. The 2D mass spectrum exhibits a clear autocorrelation line, whose signals correspond with the precursors in correlation with their own cyclotron frequency. The 2D mass spectrum shows also fragment ions from primary CAD ions generated by IRMPD in the ICR-cell. Harmonic lines and noise lines corresponding to frequencies captured during the acquisition can also be observed. Scintillation noise was minimized using the urQRd algorithm, whose de-noising rank was sufficient for a clear visualization of the 2D mass spectrum.

Table S.2: CAD fragments assigned to the autocorrelation line of the CAD/IRMPD MS/2D MS experiment on CaM.

| **Precursor ion** | **Charge** | **Theoretical m/z** | **Observed m/z** | **Error [ppm]** | **Internal** |
| --- | --- | --- | --- | --- | --- |
| y4 | 1 | 450.238082 | 450.237252 | 1.84 |  |
| AELQD | 1 | 557.256570 | 557.256391 | 0.32 | 46-50 |
| b5 | 1 | 571.272220 | 571.272281 | -0.11 |  |
| y5 | 1 | 581.278567 | 581.277702 | 1.49 |  |
| DMINE | 1 | 603.244291 | 603.245342 | -1.74 | 50-54 |
| NPTEAE | 1 | 642.272949 | 642.272972 | -0.04 | 42-47 |
| NEVDAD/ADIDGDG | 1 | 644.252214 | 644.252256 | -0.07 |  |
| b6-H2O | 1 | 682.304249 | 682.303826 | 0.62 |  |
| GQVNYE | 1 | 691.304600 | 691.306375 | -2.57 | 134-139 |
| ? | 2 |  | 702.312273 |  |  |
| y6 | 1 | 709.337145 | 709.326292 | 15.30 |  |
| y12 | 2 | 745.836347 | 745.836405 | -0.08 |  |
| NYEEFV/VNYEEF | 1 | 782.335500 | 782.335278 | 0.28 |  |
| y13 | 2 | 795.370554 | 795.370698 | -0.18 |  |
| AELQDMI | 1 | 801.381119 | 801.381476 | -0.45 | 46-52 |
| y7 | 1 | 808.405559 | 808.404651 | 1.12 |  |
| IREADID | 1 | 813.344700 | 813.343908 | 0.97 | 125-131 |
| EAELQDM/DMINEVD | 1 | 817.339649 | 817.339823 | -0.21 |  |
| DGNGTIDF/GQVNYEE | 1 | 820.347200 | 820.348384 | -1.44 |  |
| GQNPTEAE | 1 | 827.353000 | 827.352437 | 0.68 |  |
| b7 | 1 | 829.357408 | 829.357339 | 0.08 |  |
| QDMINEV | 1 | 830.371300 | 830.372007 | -0.85 | 49-55 |
| ? | 2 |  | 835.891340 |  |  |
| LQDMINE/ELQDMIN | 1 | 844.386933 | 844.387624 | -0.82 |  |
| ? | 1 |  | 884.399641 |  |  |
| LQDMINEV | 1 | 943.455347 | 943.454436 | 0.97 | 48-55 |
| b16 | 2 | 946.949267 | 946.950328 | -1.12 |  |
| y8 | 1 | 955.473973 | 955.473291 | 0.71 |  |
| ? | 6 |  | 961.967999 |  |  |
| b44-H2O | 5 | 968.276100 | 968.275281 | 0.85 |  |
| b44 | 5 | 971.878200 | 971.876769 | 1.47 |  |
| ELQDMINE | 1 | 973.429527 | 973.430300 | -0.79 | 47-54 |
| DGDGQVNYE | 1 | 978.379900 | 978.379207 | 0.71 | 131-139 |
| b17-H2O | 2 | 981.460000 | 981.463402 | -3.47 |  |
| b45-H2O | 5 | 994.084600 | 994.084355 | 0.25 |  |
| b45 | 5 | 997.686713 | 997.685926 | 0.79 |  |
| NPTEAELQD | 1 | 998.442535 | 998.451681 | -9.16 | 42-50 |
| ? | 6 |  | 1002.483911 |  |  |
| ? | 6 |  | 1005.483795 |  |  |
| b27-H2O | 3 | 1008.476739 | 1008.490591 | -13.74 |  |
| b46 | 5 | 1011.894136 | 1011.894757 | -0.61 |  |
| ? | 6 |  | 1018.993706 |  |  |
| ? | 6 |  | 1021.995236 |  |  |
| b47-H2O | 5 | 1034.100542 | 1034.103828 | -3.18 |  |
| b47 | 5 | 1037.702655 | 1037.702269 | 0.37 |  |
| b37 | 4 | 1040.261048 | 1040.261333 | -0.27 |  |
| AELQDMINE/EAELQDMIN | 1 | 1044.466641 | 1044.469617 | -2.85 | 46-54/45-53 |
| b18 | 2 | 1047.007300 | 1047.007948 | -0.62 |  |
| ?-H2O | 6 |  | 1050.006841 |  |  |
| ? | 6 |  | 1053.007879 |  |  |
| b48-H2O | 5 | 1056.717355 | 1056.715775 | 1.49 |  |
| b48 | 5 | 1060.319468 | 1060.319639 | -0.16 |  |
| b38 | 4 | 1062.019055 | 1062.021245 | -2.06 |  |
| b9 | 1 | 1070.500050 | 1070.507647 | -7.10 |  |
| b29-H2O | 3 | 1075.841858 | 1075.843873 | -1.87 |  |
| b49-NH3 | 5 | 1082.525873 | 1082.527639 | -1.63 |  |
| y9 | 1 | 1084.516567 | 1084.513810 | 2.54 |  |
| b49 | 5 | 1085.931183 | 1085.934533 | -3.08 |  |
| y19 | 2 | 1087.990283 | 1088.002125 | -10.88 |  |
| b39 | 4 | 1090.290071 | 1090.290962 | -0.82 |  |
| b40 | 4 | 1104.545437 | 1104.547310 | -1.70 |  |
| b50 | 5 | 1108.936572 | 1108.937801 | -1.11 |  |
| b19 | 2 | 1120.541520 | 1120.541898 | -0.34 |  |
| b51 | 5 | 1135.144669 | 1135.145339 | -0.59 |  |
| b41 | 4 | 1136.560081 | 1136.557279 | 2.47 |  |
| b10 | 1 | 1141.537164 | 1141.538770 | -1.41 |  |
| b52-H2O | 5 | 1154.159369 | 1154.158732 | 0.55 |  |
| b52 | 5 | 1157.761482 | 1157.761959 | -0.41 |  |
| b42 | 4 | 1165.070813 | 1165.056253 | 12.50 |  |
| b53 | 5 | 1180.570067 | 1180.567182 | 2.44 |  |
| b54 | 5 | 1206.378586 | 1206.371870 | 5.57 |  |
| b55 | 5 | 1226.192269 | 1226.185247 | 5.73 |  |
| b45 | 4 | 1246.856573 | 1246.859496 | -2.34 |  |
| b47 | 4 | 1296.876500 | 1296.874042 | 1.89 |  |
| b48 | 4 | 1325.147516 | 1325.160317 | -9.66 |  |
| y93 | 8 | 1332.748100 | 1332.746166 | 1.45 |  |
| b51 | 4 | 1418.679017 | 1418.676242 | 1.96 |  |
| b52 | 4 | 1446.950033 | 1446.940278 | 6.74 |  |
| NPTEAELQDMINE | 1 | 1485.652606 | 1485.642414 | 6.86 | 42-54 |
| EEEIREAFRVFD | 1 | 1521.7332 | 1521.721712 | 7.55 | 82-93 |
| NPTEAELQDMINEV | 1 | 1584.721020 | 1584.716534 | 2.83 | 42-55 |
| DGDGQVNYEEFVQM | 1 | 1612.658400 | 1612.657381 | 0.63 | 131-144 |
| b29 | 2 | 1622.264400 | 1622.267799 | -2.10 |  |
| GDGQVNYEEFVQMM | 1 | 1628.672000 | 1628.678904 | -4.24 | 132-145 |
| PTEAELQDMINEVDA | 1 | 1656.742100 | 1656.731559 | 6.36 | 43-57 |
| GQNPTEAELQDMINE | 1 | 1670.732648 | 1670.731305 | 0.80 | 40-54 |
| NPTEAELQDMINEVD | 1 | 1699.748000 | 1699.743736 | 2.51 | 42-56 |
| QNPTEAELQDMINEV | 1 | 1712.779598 | 1712.803080 | -13.71 | 41-55 |
| DGDGQVNYEEFVQMM-H2O | 1 | 1725.688300 | 1725.672986 | 8.87 | 131-145 |
| GDGQVNYEEFVQMMT | 1 | 1729.719600 | 1729.719975 | -0.22 | 132-146 |
| DGDGQVNYEEFVQMM | 1 | 1743.698905 | 1743.698595 | 0.18 | 131-145 |
| GQNPTEAELQDMINEV-NH3 NPTEAELQDMINEVDA-H2O | 1 | 1752.774500 | 1752.783885 | -5.35 |  |
| GQNPTEAELQDMINEV | 1 | 1769.801062 | 1769.801708 | -0.36 | 40-55 |
| DGDGQVNYEEFVQMMT | 1 | 1844.746600 | 1844.742435 | 2.26 | 131-146 |
| GQNPTEAELQDMINEVD | 1 | 1884.828006 | 1884.828505 | -0.26 | 40-56 |
| ? | 1 |  | 1892.889751 |  |  |
| TDEEVDEMIREADIDGD-2H2O | 1 | 1897.775600 | 1897.775904 | -0.16 | 117-133 |
| GQNPTEAELQDMINEVDA | 1 | 1955.865120 | 1955.874241 | -4.66 | 40-57 |
| DIDGDGQVNYEEFVQMM | 1 | 1971.809900 | 1971.799666 | 5.19 | 129-145 |
|  |  | Average error [ppm] | | 0.10 |  |
|  |  | STD | | 3.11 |  |

#### CAD/ECD MS/2D MS of CaM

The 2D mass spectrum was acquired with 2k data-points in the vertical dimensions and 1M data-points (16 bit) in the horizontal dimension. The resulting vertical and horizontal resolving powers are respectively RP_v_ ≈ 600 and RP_h_ ≈ 100000, reported at *m/z* 400. The total resolving power was RP_2D_ ≈ 60 M, reported at the same *m/z*. Calibration was performed internally during instrumentation setup, using a standard mixture. The 2D mass spectrum of Figure 2.e shows a clear autocorrelation line and peaks corresponding to fragmentation of the precursor ions. Several harmonic lines and electron-capture lines can be observed. Electron-capture lines are highlighted in the spectrum, showing all the precursors that capture one or more electron during the fragmentation period with ECD in the ICR-cell. Scintillation noise was reduced through the urQRd algorithm to a level that made possible to analyze and interpret the 2D mass spectrum.

The autocorrelation line was extracted and calibrated internally using theoretical *m/z* of the recognized fragments. Several *b/y* ions, internal fragment ions and neutral losses could be assigned, with an average mass accuracy of 2.42±4.66 ppm. Percentages of the assigned ions are reported in Figure S.3 below, together with a complete list of the correct peak assignments for the autocorrelation line of the CAD/ECD MS/2D MS of CaM in denaturing conditions. Although the autocorrelation lines were obtained with the same CAD and ion transfer parameters for the two experiments they are mostly similar for the assigned fragments, the most common of which could be found for both the analyses, but they differ in signal intensity, and some different precursors can be found in each of them (see tables S.2 and S.3). The cleavage coverage obtained for the CAD fragmentation of CaM, calculated on the autocorrelation line considering cleavages due to internal fragmentation, is of ~31%.

Figure 2.f shows the extraction of a fragment ion scan. Ion *b*_54_^5+^ was chosen as example to highlight the different fragmentation patterns obtained with the CAD/IRMPD and CAD/ECD experiments. Charge-reduced species corresponding to the capture of one and two electrons, accompanied by several side-chain losses, can be observed in the mass spectrum in Figure 2.f, together with *c* ions: these are typical ions originated with ion activation by electron-capture dissociation. Many unassigned fragments are also present. Direct comparison of the autocorrelation fragment ion scans of Figure 2.c and Figure 2.f shows the different fragmentation patterns exhibited by the CAD-fragment *b_54_^5+^* when subjected to IRMPD or ECD respectively. They demonstrated that CAD/IRMPD and CAD/ECD MS/2D MS experiments are indeed complementary. Horizontal fragment ion scans were extracted for all the successfully assigned precursors, and their fragmentation patterns assigned and used to calculate the cleavage coverage of CaM with the CAD/ECD MS/2D MS experiment. The value of this cleavage coverage is ~41%, calculated considering also the cleavages due to internal fragmentation, and showed in the cleavage coverage map of Figure 3.

Table S.3: CAD fragments assigned to the autocorrelation line of the CAD/ECD MS/2D MS experiment on CaM.

| **Fragment ion** | **Charge** | **Theoretical m/z** | **Observed m/z** | **Err [ppm]** | **Internal** |
| --- | --- | --- | --- | --- | --- |
| PTEAE | 1 | 528.23 | 528.229218 | 1.48 | 43-47 |
| NYEE | 1 | 536.1987 | 536.197937 | 1.42 | 37-40 |
| AELQD | 1 | 557.25657 | 557.256261 | 0.55 | 46-50 |
| b5 | 1 | 571.27222 | 571.271575 | 1.13 |  |
| y5 | 1 | 581.278567 | 581.278635 | -0.12 |  |
| PTEAEL | 1 | 641.3141 | 641.314357 | -0.40 | 43-48 |
| NPTEAE | 1 | 642.272949 | 642.273193 | -0.38 | 42-47 |
| NEVDAD/ADIDGDG | 1 | 644.252214 | 644.253124 | -1.41 |  |
| b6-H2O | 1 | 682.304249 | 682.306059 | -2.65 |  |
| y11 | 2 | 688.8149 | 688.814236 | 0.96 |  |
| y6-NH3 | 1 | 692.310596 | 692.312884 | -3.31 |  |
| ? | 1 | 702.313161 | 702.313380 | -0.31 |  |
| y6 | 1 | 709.337145 | 709.337596 | -0.64 |  |
| y12 | 2 | 745.836347 | 745.842473 | -8.21 |  |
| PTEAELQ | 1 | 769.372663 | 769.376470 | -4.95 | 43-49 |
| NYEEFV/VNYEEF | 1 | 782.3355 | 782.337189 | -2.16 |  |
| y13 | 2 | 795.370554 | 795.371245 | -0.87 |  |
| AELQDMI | 1 | 801.381119 | 801.384718 | -4.49 | 46-52 |
| y7 | 1 | 808.405559 | 808.410600 | -6.24 |  |
| DGNGYISAA-2H2O ? | 1 |  | 813.349172 |  |  |
| EAELQDM/DMINEVD | 1 | 817.339649 | 817.341385 | -2.12 |  |
| GQNPTEAE | 1 | 827.353 | 827.355943 | -3.56 | 40-47 |
| b7 | 1 | 829.357408 | 829.361369 | -4.78 |  |
| ? | 2 | 835.894444 | 835.896468 | -2.42 |  |
| LQDMINE/ELQDMIN | 1 | 844.386933 | 844.391137 | -4.98 |  |
| b15 | 2 | 873.4151 | 873.416881 | -2.04 |  |
| QNPTEAEL/NPTEAELQ | 1 | 883.415591 | 883.418610 | -3.42 |  |
| ? | 1 | 884.400036 | 884.404264 | -4.78 |  |
| QVNYEEF/NYEEFVQ | 1 | 910.3941 | 910.396928 | -3.11 |  |
| AELQDMIN | 1 | 915.424047 | 915.426880 | -3.09 | 46-53 |
| ? | 6 |  | 921.452225 |  |  |
| b42-H2O | 5 | 928.656000 | 928.660891 | -5.27 |  |
| b42 | 5 | 932.2581 | 932.261479 | -3.62 |  |
| LQDMINEV | 1 | 943.455347 | 943.454829 | 0.55 | 48-55 |
| b16 | 2 | 946.949267 | 946.948885 | 0.40 |  |
| y8 | 1 | 955.473973 | 955.479018 | -5.28 |  |
| ? | 6 |  | 961.970093 |  |  |
| ? | 5 |  | 962.805820 |  |  |
| b44-H2O | 5 | 968.2761 | 968.278839 | -2.83 |  |
| b44 | 5 | 971.8782 | 971.881198 | -3.08 |  |
| DGDGQVNYE | 1 | 978.3799 | 978.382787 | -2.95 | 31-39 |
| b17-H2O | 2 | 981.46 | 981.458885 | 1.14 |  |
| b45-2H2O | 5 | 990.4825 | 990.481702 | 0.81 |  |
| b45-H2O | 5 | 994.0846 | 994.088114 | -3.53 |  |
| b45 | 5 | 997.686713 | 997.690910 | -4.21 |  |
| NPTEAELQD | 1 | 998.442535 | 998.445114 | -2.58 | 42-50 |
| ? | 6 |  | 1002.486214 |  |  |
| b27-H2O | 3 | 1008.47674 | 1008.477937 | -1.19 |  |
| b46 | 5 | 1011.89414 | 1011.897645 | -3.47 |  |
| y18 | 2 | 1031.44825 | 1031.446932 | 1.28 |  |
| b47-H2O | 5 | 1034.10054 | 1034.105283 | -4.58 |  |
| b37-H2O | 4 | 1035.75841 | 1035.764857 | -6.23 |  |
| b47 | 5 | 1037.70265 | 1037.705807 | -3.04 |  |
| b37 | 4 | 1040.26105 | 1040.265825 | -4.59 |  |
| AELQDMINE/EAELQDMIN | 1 | 1044.46664 | 1044.471924 | -5.06 |  |
| ? | 6 |  | 1050.012127 |  |  |
| ? | 6 |  | 1055.475919 |  |  |
| b48-H2O | 5 | 1056.71735 | 1056.720151 | -2.65 |  |
| b38-H2O | 4 | 1057.51641 | 1057.522508 | -5.76 |  |
| b48 | 5 | 1060.31947 | 1060.322963 | -3.30 |  |
| b38 | 4 | 1062.01905 | 1062.021917 | -2.70 |  |
| y9-H2O | 1 | 1066.5 | 1066.504788 | -4.49 |  |
| b9 | 1 | 1070.50005 | 1070.501031 | -0.92 |  |
| ? | 2 | 1076.19 | 1076.178094 | 11.06 |  |
| b39-2H2O | 4 | 1081.28479 | 1081.288302 | -3.25 |  |
| b49-H2O | 5 | 1082.32907 | 1082.326989 | 1.92 |  |
| y9 | 1 | 1084.51657 | 1084.520372 | -3.51 |  |
| b39-H2O | 4 | 1085.78743 | 1085.791275 | -3.54 |  |
| b49 | 5 | 1085.93118 | 1085.933687 | -2.31 |  |
| b39 | 4 | 1090.29007 | 1090.294277 | -3.86 |  |
| ? | 4 |  | 1093.798130 |  |  |
| b40-H2O | 4 | 1100.0428 | 1100.048668 | -5.34 |  |
| b40 | 4 | 1104.54544 | 1104.549989 | -4.12 |  |
| b50 | 5 | 1108.93657 | 1108.941953 | -4.85 |  |
| b19 | 2 | 1120.54152 | 1120.542982 | -1.30 |  |
| b51-H2O | 5 | 1131.54256 | 1131.546635 | -3.60 |  |
| b41-H2O | 4 | 1132.05744 | 1132.061280 | -3.39 |  |
| b51 | 5 | 1135.14467 | 1135.150301 | -4.96 |  |
| b41 | 4 | 1136.56008 | 1136.564219 | -3.64 |  |
| b10 | 1 | 1141.53716 | 1141.530251 | 6.06 |  |
| y90-H2O | 9 | 1149.3211 | 1149.332209 | -9.67 |  |
| b52-H2O | 5 | 1154.15937 | 1154.164167 | -4.16 |  |
| b52 | 5 | 1157.76148 | 1157.766645 | -4.46 |  |
| b42-H2O | 4 | 1160.56817 | 1160.575753 | -6.53 |  |
| b42-H2O deamidated? | 4 | 1160.81418 | 1160.821279 | -6.12 |  |
| y90 | 9 | 1164.1031 | 1164.107863 | -4.09 |  |
| b42 | 4 | 1165.07081 | 1165.075349 | -3.89 |  |
| y92-NH3 | 9 | 1170.1043 | 1170.111609 | -6.25 |  |
| y92 | 9 | 1171.9961 | 1172.001147 | -4.31 |  |
| b53 | 5 | 1180.57007 | 1180.580754 | -9.05 |  |
| y93-H2O | 9 | 1182.7757 | 1182.778857 | -2.67 |  |
| y93 | 9 | 1184.7769 | 1184.780062 | -2.67 |  |
| y96-2NH3 | 9 | 1219.0103 | 1219.016379 | -4.99 |  |
| y96-NH3 | 9 | 1220.9021 | 1220.911673 | -7.84 |  |
| b55-H2O | 5 | 1222.59016 | 1222.574650 | 12.68 |  |
| b55 | 5 | 1226.19227 | 1226.200988 | -7.11 |  |
| PTEAELQDMIN/NPTEAELQDMI | 1 | 1242.56708 | 1242.554454 | 10.16 |  |
| b45 | 4 | 1246.85657 | 1246.861835 | -4.22 |  |
| ? | 2 |  | 1247.279620 |  |  |
| b57 | 5 | 1263.40508 | 1263.383515 | 17.07 |  |
| b46 | 4 | 1264.61585 | 1264.621188 | -4.22 |  |
| b47 | 4 | 1296.8765 | 1296.878982 | -1.91 |  |
| y91-2NH3 | 8 | 1305.2334 | 1305.246159 | -9.77 |  |
| y91-NH3 | 8 | 1307.3617 | 1307.374492 | -9.78 |  |
| y91 | 8 | 1309.49007 | 1309.503065 | -9.92 |  |
| y92 | 8 | 1318.36971 | 1318.371954 | -1.70 |  |
| b48 | 4 | 1325.14752 | 1325.118833 | 21.64 |  |
| y93-NH3 | 8 | 1330.6198 | 1330.628293 | -6.38 |  |
| y93 | 8 | 1332.7481 | 1332.753610 | -4.13 |  |
| y94-H2O | 8 | 1342.88031 | 1342.886148 | -4.35 |  |
| y96 | 8 | 1375.51732 | 1375.517607 | -0.21 |  |
| b50 | 4 | 1385.9189 | 1385.928876 | -7.20 |  |
| b51 | 4 | 1418.67902 | 1418.684627 | -3.95 |  |
| ? | 7 |  | 1439.973450 |  |  |
| b52 | 4 | 1446.95003 | 1446.963298 | -9.17 |  |
| ? | 11 | 1462.05339 | 1462.052908 | 0.33 |  |
| PTEAELQDMINEV | 1 | 1470.67809 | 1470.678523 | -0.29 | 43-55 |
| y89 | 7 | 1471.83784 | 1471.834649 | 2.17 |  |
| y90-3NH3 | 7 | 1475.119 | 1475.122049 | -2.07 |  |
| y90-NH3 | 7 | 1477.5514 | 1477.554798 | -2.30 |  |
| y90 | 7 | 1479.98377 | 1479.980926 | 1.92 |  |
| FDKDGNGYISAAEL | 1 | 1481.6907 | 1481.702091 | -7.69 |  |
| NPTEAELQDMINE | 1 | 1485.65261 | 1485.658412 | -3.91 | 42-54 |
| y12 | 1 | 1490.66542 | 1490.671395 | -4.01 |  |
| b54 | 4 | 1507.72141 | 1507.723659 | -1.49 |  |
| ? | 6 | 1528.61 | 1528.442091 |  |  |
| y94 | 7 | 1537.1494 | 1537.147680 | 1.12 |  |
| NPTEAELQDMINEV | 1 | 1584.72102 | 1584.718120 | 1.83 | 42-55 |
| b59-3H2O | 4 | 1608.50871 | 1608.514836 | -3.81 |  |
| ? | 1 |  | 1612.727173 |  |  |
| GQNPTEAELQDMINE | 1 | 1670.73265 | 1670.739351 | -4.01 | 40-54 |
| NPTEAELQDMINEVD | 1 | 1699.74374 | 1699.752237 | -5.00 | 42-56 |
| GDGQVNYEEFVQMMT | 1 | 1729.71998 | 1729.726696 | -3.89 | 132-146 |
| DGDGQVNYEEFVQMM | 1 | 1743.69891 | 1743.706542 | -4.38 | 131-145 |
| y30 | 2 | 1746.268009 | 1746.27271 | -2.69 |  |
| GQNPTEAELQDMINEV | 1 | 1769.80106 | 1769.801488 | -0.24 | 40-55 |
| PTEAELQDMINEVDAD | 1 | 1771.76909 | 1771.765980 | 1.76 | 43-58 |
| GQNPTEAELQDMINEVD | 1 | 1884.82801 | 1884.842019 | -7.43 | 40-56 |
| AELQDMINEVDADGNGTI | 1 | 1886.8436 | 1886.825341 | 9.68 | 46-63 |
| QNPTEAELQDMINEVDA | 1 | 1898.8436 | 1898.838924 | 2.46 | 41-57 |
| DMINEVDADGNGTIDFPE | 1 | 1933.812 | 1933.812290 | -0.15 | 50-67 |
| y17 | 1 | 1946.86228 | 1946.864204 | -0.99 |  |
| GQNPTEAELQDMINEVDA | 1 | 1956.86392 | 1956.880434 | -8.44 | 40-57 |
| DIDGDGQVNYEEFVQMM | 1 | 1971.79967 | 1971.816153 | -8.36 | 129-145 |


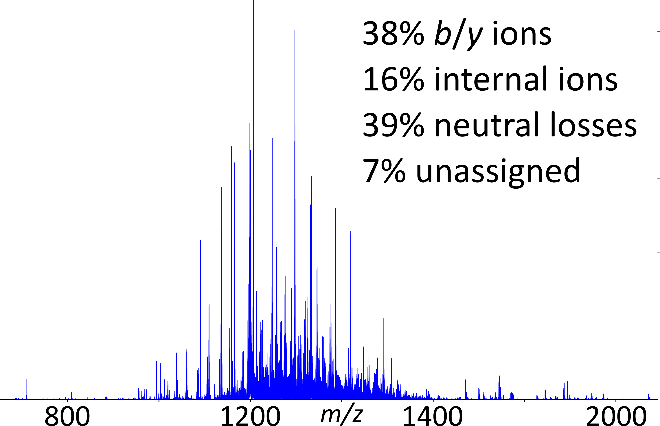


Figure S.3: Autocorrelation line extracted from the CAD/ECD MS/2D MS spectrum of CaM.

### Parameters for MS acquisition of Calmodulin

Table S.4: Parameters used for the acquisition of the one-dimensional and two-dimensional mass spectra of Calmodulin.

| **Parameters** | **FT-ICR MS** | | **MS/2D MS** | |
| --- | --- | --- | --- | --- |
|  | **MS** | **CAD MS/MS** | **CAD/IRMPD** | **CAD/ECD** |
| Acquisition mode | | | | |
| Acquisition size [data points, 16 bits] | 4M | 4M | 512k | 512k |
| Horizontal low mass cut-off [m/z] | 147.41 | 147.41 | 368.2 | 368.2 |
| Horizontal high mass cut-off [m/z] | 3000 | 3000 | 3000 | 3000 |
| Transient length, t2 [s] | 1.6777 | 1.6777 | 0.2097 | 0.2097 |
| Ion accumulation time [s] | 0.2 | 0.2 | 0.2 | 0.2 |
| Ion cooling time [s] | 0.1 | 0.1 | 0.1 | 0.1 |
| **Source (ESI)** | | | | |
| Flow rate [uL/h] | 70 | 70 | 70 | 70 |
| Capillary voltage [V] | -4800 | -4800 | -4800 | -4800 |
| EP Off-set [v] | -500 | -500 | -500 | -500 |
| Nebulizer Pressure [bar] | 1.2 | 1.2 | 1.2 | 1.2 |
| Capillary exit [V] | 200 | 200 | 200 | 200 |
| Deflector plate [V] | 220 | 220 | 220 | 220 |
| Funnel [V] | 150 | 150 | 150 | 150 |
| Skimmer [V] | 10 | 10 | 10 | 10 |
| Funnel RF [V_pp_] | 200 | 200 | 200 | 200 |
| **Collision cell** | | | | |
| Collision voltage [V] | -4 | -13.5 | -13.5 | -13.5 |
| DC extract bias [V] | 0.6 | 0.6 | 0.6 | 0.6 |
| RF frequency [MHz] | 2 | 2 | 2 | 2 |
| Collision RF Amplitude [Vpp] | 1400 | 1400 | 1400 | 1400 |
| **Octopole** | | | | |
| Frequency [MHz] | 2 | 2 | 2 | 2 |
| RF Amplitude [Vpp] | 1400 | 1400 | 1400 | 1400 |
| **Transfer optics** | | | | |
| Time of Flight [s] | 0.001 | 0.001 | 0.0012 | 0.001 |
| Frequency [MHz] | 4 | 4 | 4 | 4 |
| RF Amplitude [Vpp] | 350 | 350 | 350 | 350 |
| **ICR-cell** | | | | |
| Transfer exit Lens [V] | -15 | -15 | -15 | -15 |
| Analyzer entrance [V] | -8 | -8 | -8 | -8 |
| Side kick [V] | 5 | 12 | 10 | 5 |
| Side kick offset [V] | -1.5 | -2.3 | -2.9 | -1.5 |
| Front Trap plate [V] | 0.51 | 0.51 | 0.69 | 0.91 |
| Back Trap plate [V] | 0.55 | 0.55 | 0.74 | 0.96 |
| Sweep Excitation power, P3 [%] | 16 | 16 | 16 | 16 |
| **In-cell manipulations** | | | | |
|  | **multi-CHEF** | | **2D MS pulse programme** | |
| t1 [s] | n/a | n/a | 1.00E-06 | 1.00E-06 |
| t1 increment [s] | n/a | n/a | 1.00E-06 | 1.00E-06 |
| P1 and P2 pulse length [s] | n/a | n/a | 4.00E-07 | 4.00E-06 |
| P1 and P2 excitation power [Db] | n/a | n/a | 10.42 | 10.42 |
| Loops | n/a | n/a | 2048 | 2048 |
| **In-cell Fragmentation** | | | | |
| Laser Power (25W) [%] | / | / | 50 | / |
| IR pulse length [s] | / | / | 0.16 | / |
| ECD pulse length [s] | / | / | / | 0.016 |
| ECD bias | / | / | / | 1.5 |
| ECD Extraction lens | / | / | / | 10 |
